# Comprehensive pharmacogenomics profiling of the Serbian population

**DOI:** 10.3389/fphar.2025.1553536

**Published:** 2025-03-17

**Authors:** Marina Jelovac, Djordje Pavlovic, Biljana Stankovic, Nikola Kotur, Bojan Ristivojevic, Sonja Pavlovic, Branka Zukic

**Affiliations:** Group for Molecular Biomedicine, Department of Human Molecular Genetics and Genomics, Institute of Molecular Genetics and Genetic Engineering, University of Belgrade, Belgrade, Serbia

**Keywords:** pharmacogenomics, bioinformatics, population genetics, personalized medicine, high-throughput sequencing

## Abstract

**Background:**

Pharmacogenomics offers a possibility of anticipating drug response based on individuals’ genetic profiles and represents a step toward implementation of personalized treatment through routine genetic testing. Development of highthroughput sequencing technologies aided identification and interpretation of variants in many pharmacogenes simultaneously. Nonetheless, the integration of pharmacogenomics into clinical practice is arduous, partly due to insufficient knowledge of ethnic pharmacogenetic data. The aim of our study was to assemble the most comprehensive pharmacogenomics landscape of the Serbian population so far.

**Methods:**

We used genomic data of 881 individuals from Serbia obtained by clinical and whole exome sequencing. Raw sequencing files were processed using an in-house pipeline for alignment and variant calling. For annotation of pharmacogenetics star alleles and determination of phenotypes, we used the PharmCAT and Stargazer tools. Star allele and phenotype frequencies were calculated and compared to worldwide and European populations. Population differentiation was presented through calculation of Wright’s fixation index.

**Results:**

Our results showed that population differentiation was the highest between the Serbian and the worldwide population. In the Serbian population, the most relevant pharmacogenes in terms of star allele frequencies and actionable phenotypes were *CYP2B6, NAT2, SLCO1B1, UGT1A1* and *VKORC1*, that had significantly different distribution compared to other European populations.

**Conclusion:**

In conclusion, significant differences in frequencies of pharmacogenetic phenotypes that influence response to several drug categories including statins and antidepressants indicate that inclusion of data relevant for drug response to genetic reports would be beneficial in the Serbian population. Implementation of pharmacogenetic testing could be achieved through analysis of clinical and whole exome sequencing data.

## 1 Introduction

With a goal of maximizing drug efficacy and safety according to the genetic profile of each patient, pharmacogenomics (PGx) represents an aspect of precision medicine that has greatly benefited from technological advancements (Ng et al., 2017). Expansion and cost-effectiveness of next-generation sequencing (NGS) facilitated shifting from a one-size-fits-all approach towards precision medicine, which focuses on patients’ unique genetic signatures (Morash et al., 2018). Clinical implementation of pharmacogenomics is already being performed, mostly for single genes or panels of variants with a well-described effect on drug response (Tafazoli et al., 2021; Russell et al., 2021). Clinical implementation relies on drug prescribing guidelines provided by pharmacogenomics associations, most notably the Clinical Pharmacogenetics Implementation Consortium (CPIC) and the Dutch Pharmacogenetics Working Group (DPWG). CPIC and DPWG provide a scoring system and level of evidence associated with each gene-drug pair. A similar system for ranking pharmacogenomic evidence is provided by the Pharmacogenomics Knowledge Base (PharmGKB), which is the most comprehensive resource for pharmacogenomics.

Although a considerable amount of data concerning gene effect on drug response is already available, implementation of PGx knowledge into clinical practice is still scarce (Kabbani et al., 2023). Next-generation sequencing is widely used for molecular diagnostics and other clinical analyses, leaving a plethora of genomic data available for identification and interpretation of genetic variants and haplotypes, or star alleles, that influence drug response (Ji et al., 2018). Constant increase of sequencing power and decrease in cost of sequencing offer possibilities for fast and cost-effective, as well as preemptive and comprehensive pharmacogenomics profiling.

Furthermore, interethnic genome landscape differences are important for the implementation of PGx into clinical settings (Karamperis et al., 2024; Zhou and Lauschke, 2022). The genomic landscape of the Serbian population was previously described, showing unique features in this population (Drljaca et al., 2021; Paschou et al., 2014). The data remaining after diagnostic sequencing has a great potential for characterizing the pharmacogenomic profile of a specific population (Nunez-Torres et al., 2023; Hočevar, Maver, and Peterlin, 2019; Zhang and Lauschke, 2019). Meticulous depiction of subpopulations brings the opportunity for better risk stratification, leading to the development of population-specific genotyping strategies (Karamperis et al., 2024; Zhou and Lauschke, 2022; Wright et al., 2018).

Technological advancement elicited a trend of population-wide precision medicine, with many Western Balkan countries reporting specific pharmacogenomic profiles (Hočevar, Maver, and Peterlin, 2019; Proietti et al., 2023; Jmel et al., 2024; Matišić et al., 2023; Perović et al., 2024; Velizarova et al., 2022). In Serbia, a considerable number of studies addressing response to different drugs were performed, including treatment of pediatric acute lymphoblastic leukemia (Dokmanovic et al., 2006; Kotur et al., 2020), inflammatory bowel disease (Stankovic et al., 2020; Jojic et al., 2003), rheumatoid arthritis (Jančić et al., 2013; Jančić et al., 2015) etc. However, a comprehensive understanding of the pharmacogenomic landscape in the Serbian population remains lacking. Re-use of the large amount of NGS data generated through genetic testing of patients with suspected rare diseases presents an opportunity to assess population-level genomic and pharmacogenomic characteristics in Serbia (Gemovic and Veljkovic, 2023; Andjelkovic et al., 2024; Stankovic et al., 2020).

In this study, we aimed to investigate the pharmacogenomic landscape of the Serbian population by analyzing over 50 pharmacogenes using large-scale NGS data and bioinformatics tools tailored for PGx. The focus was to determine the frequency of star alleles and PGx phenotypes and to compare our findings with the worldwide and European population, aiming to identify any unique PGx characteristics of the Serbian population. Another goal of our study was to assess the feasibility of using CES and WES for the detection of PGx variants relevant for drug response and therapy optimization.

## 2 Materials and methods

### 2.1 Subjects and exome sequencing

The genetic data of 881 individuals from the Serbian population were used in this study. DNA was isolated from blood samples, which were subsequently sequenced either with clinical or whole exome panels. Samples were sequenced over a 2-year period, from 2023 to 2024. For the presented study, data of all patients were de-identified, with subject sex being the only retained information relevant for *G6PD* genotyping. No phenotype data was available. The study and use of genomic data was approved by the Ethics Committee of the Institute of Molecular Genetics and Genetic Engineering, University of Belgrade (O-EO-046/2023).

The majority of samples (768 samples) underwent clinical exome sequencing (TruSight One Sequencing Panel, Illumina, CA, United States) and the rest of the subjects (113 samples) were sequenced using a whole exome panel (Illumina DNA Prep with Exome 2.0 Plus Enrichment, Illumina, CA, United States). The clinical exome sequencing panel covers selected exons of 4,813 genes, containing variants which have clinical relevance. Whole exome sequencing provides significantly more information, since it covers all exons and even several important intronic regions.

### 2.2 Bioinformatic analysis

Raw files obtained with NGS were processed using an in-house pipeline that follows the GATK Best Practices guidelines (Poplin et al., 2017). Mapping was performed on the hg38 reference genome assembly provided by GATK, using the bwa-mem algorithm. Alignment files obtained this way were further processed using GATK tools for marking duplicates and recalibrating base quality scores to create analysis-ready BAM files. These were then used to perform variant calling with HaplotypeCaller along with appropriate intervals to create gVCF files, which were combined into two groups: one analyzed with a clinical exome and the other with a whole exome sequencing panel. Joint genotyping and filtering was then performed on these two groups to obtain multi-sample VCF files for downstream analysis. The joint genotyping was done using GenotypeGVCFs with the added flag*--force-output-intervals* to make sure that known pharmacogenomics variants do not get missed if they have a reference value in the entire group. For this purpose we used genomic positions of all relevant pharmacogenetic variants, provided by the PharmCAT tool (Sangkuhl et al., 2020). Created multi-sample VCF files were ready to use in the PharmCAT downstream analysis that involved pre-processing, allele matching and phenotype assignment. The PharmCAT *json2tsv* python script and in-house written R script were used to extract and organize data in the multi-sample tabular file format for subsequent statistical analysis.

Additionally, single sample BAM and VCF files were used for analysis with the Stargazer v2.0 tool (Lee et al., 2019). Stargazer is also used for calling star alleles in PGx genes, but unlike PharmCAT, it can detect complex structural variants by referring to read depth for calculation of paralog-specific copy numbers. Read depth is determined by comparing depths in a gene of interest, flagged by*--target-gene*, and a preselected control gene used as a normalization factor for copy number analysis (flagged by*--control-gene*). To this end, Stargazer uses BAM files for creation of a specific GATK-DepthOfCoverage format (GDF) file in form of a table, which stores read depth information. By combining annotated VCF with a created GDF, Stargazer extracts variants in selected pharmacogenes.

As a final result, both PharmCAT and Stargazer provide a combination of 2 haplotypes or star alleles (diplotype) for each sample and for each pharmacogene. Also, for each diplotype, a specific phenotype is assigned, related to each pharmacogene.

### 2.3 Pharmacogenetic study design

Genotyping of PGx genes was performed using two different tools: PharmCAT (Sangkuhl et al., 2020) and Stargazer (Lee et al., 2019). PharmCAT is an annotation tool that uses genetic information from VCF files on 18 genes associated with CPIC and DPWG guidelines (*ABCG2, CACNA1S, CFTR, CYP2B6, CYP2C19, CYP2C9, CYP3A4, CYP3A5, CYP4F2, DPYD, G6PD, IFNL3, NUDT15, RYR1, SLCO1B1, TPMT, UGT1A1, VKORC1*), converts genome variants into PGx star alleles and interprets phenotype given the diplotype information. The output of this software is a report with genotype-based prescribing recommendations.

Stargazer can determine haplotypes in genes covered by PharmCAT as well as 39 other PGx genes (*2C cluster, ABCB1, CYP17A1, CYP19A1, CYP1A1, CYP1A2, CYP1B1, CYP26A1, CYP2A13, CYP2A6, CYP2C8, CYP2D6, CYP2E1, CYP2F1, CYP2J2, CYP2R1, CYP2S1, CYP2W1, CYP3A43, CYP3A7, CYP4A11, CYP4A22, CYP4B1, GSTM1, GSTP1, NAT1, NAT2, POR, PTGIS, SLC15A2, SLC22A2, SLCO1B3, SLCO2B1, SULT1A1, TBXAS1, UGT1A4, UGT2B7, UGT2B15, XPC*).

Since CES panel does not cover all exons, for genes lacking full coverage only whole exome data were retained, namely, for genes *2C cluster, CYP2S1, GSTM1, NUDT15* and *SLC15A2*. Moreover, some well-described PGx variants are found in intronic regions, as is the case for the *UGT1A1* TATA box short tandem repeats-related alleles. In such cases, frequencies were calculated separately for CES or WES data. Finally, for genes in which all variants are read with both CES and WES, data was gathered and summed frequencies were calculated (Supplementary Data Sheets 2–9). Table 1 shows the summary of genes represented with clinical, whole exome or both sequencing panels.

**TABLE 1:** Lists of genes for which variant frequencies were calculated using either data from clinical exome, whole exome or both sequencing panels.

Sequencing panel	Clinical exome sequencing data	Whole exome sequencing data	Whole exome sequencing data exclusively	Clinical exome + whole exome sequencing data
Number of samples	768	113	113	881
List of genes		*CYP2C cluster*		*ABCB1*
		*CYP1A2*		*ABCG2*
		*CYP2A6*		*CFTR*
		*CYP2B6*		*CYP1A1*
		*CYP2C19*		*CYP1B1*
	*CYP1A2*	*CYP2D6*		*CYP2A13*
	*CYP2A6*	*CYP2E1*		*CYP2C8*
	*CYP2B6*	*CYP2J2*		*CYP2C9*
	*CYP2C19*	*CYP2S1 *	*CYP2C* cluster	*CYP2F1*
	*CYP2D6*	*CYP3A4*	*CYP2J2*	*CYP2W1*
	*CYP2E1*	*CYP3A5*	*CYP2S1*	*CYP3A7*
	*CYP3A4*	*CYP4A22*	*CYP3A5*	*CYP3A43*
	*CYP4A22*	*DPYD*	*GSTM1*	*CYP4A11*
	*DPYD*	*GSTM1*	*IFNL3*	*CYP4B1*
	*NAT1*	*IFNL3*	*NUDT15*	*CYP4F2*
	*SLCO1B1*	*NAT1*	*PTGIS*	*CYP19A1*
	*SULT1A1*	*NUDT15*	*SLC15A2*	*CYP26A1*
	*TBXAS1*	*PTGIS*	*VKORC1*	*G6PD*
	*UGT1A1*	*SLC15A2*		*GSTP1*
	*UGT1A4*	*SLCO1B1*		*NAT2*
		*SULT1A1*		*POR*
		*TBXAS1*		*RYR1*
		*UGT1A1*		*SLC22A2*
		*UGT1A4*		*SLCO1B3*
		*VKORC1*		*SLCO2B1*
				*TPMT*
				*UGT2B7*
				*UGT2B15*
				*XPC*

### 2.4 Population genetic comparative study and statistical analysis

All statistical analysis were performed in R software v.4.3.0.

Quality control of genotyping data was done using HardyWeinberg package (Graffelman, 2015). For each star allele Hardy-Wienberg equilibrium (HWE) was tested by comparing the number of carriers of the considered allele versus all others.

The population pharmacogenomics study compared star allele frequencies in the Serbian population with those in the worldwide, European and Croatian populations. Information on star allele frequencies in the worldwide and European populations defined by single variants was retrieved from the CPIC and the gnomAD (Chen et al., 2024) databases. However, frequencies of star alleles representing haplotypes defined by multiple variants could not be directly obtained from the gnomAD or CPIC databases. Instead, these haplotype frequencies were determined using the LDpair Tool (Machiela and Chanock, 2015), which provides frequencies for variants in linkage disequilibrium based on data from the 1000 Genomes Project. For the Croatian population, frequencies of variants in PGx genes were extracted from previously published population study (Matišić et al., 2023) and compared with frequencies in the Serbian population. All frequency comparisons in this study were performed using the Chi-squared test, provided that the expected frequency in each cell of the 2 × 2 table was at least 5. Otherwise, Fisher’s Exact Test was used. Wright’s fixation index (Fst) was used as a measure of population similarity. Calculation of Fst was performed with *bigsnpr* (Privé et al., 2018) package. Both Stargazer and PharmCAT provide predicted phenotypes for star alleles in actionable pharmacogenes. Predicted phenotypes that were detected in the Serbian population were compared with European subpopulations (1,000 Genome Project Data - Central European, Finnish, Great Britain, Iberian population in Spain and Tuscans from Italy) (Sherman, Claw, and Lee, 2024) using Fisher’s Exact or Chi-squared test. Bonferroni correction was used to adjust the statistical significance levels for multiple tests in order to reduce the number of false positive results. The correction was applied by dividing 0.05 by the number of pharmacogenes analyzed in population comparisons tests.

## 3 Results

### 3.1 Quality control and Hardy-Weinberg equilibrium

Clinical exome samples achieved a mean target coverage of 90×, while whole exome samples achieved a mean target coverage of 73×, with 92% and 86% of all positions achieving depth over 30×, respectively (Supplementary Data Sheet 10).

HWE was used as a measure of genotyping quality. Based on the number of tested pharmacogenes, the level of significance was determined by dividing 0.05 with the number of pharmacogenes with at least 2 different alleles. Adjusted p values were <0.001 (0.05/47), <0.0009 (0.05/54) and <0.002 (0.05/30) for CES, WES or the combination of both panels respectively. Pharmacogenes that harbored variants deviating from HWE were withdrawn from subsequent analysis (Supplementary Table S1; Supplementary Data Sheet 1). If one variant was out of HWE when clinical exome panel was used, but was in HWE if whole exome sequencing was performed, results from whole exome sequencing were retained. Only reference alleles were found in *CACNA1S, CYP17A1* and *CYP2R1*, therefore these pharmacogenes were excluded from further comparisons as well (Supplementary Table S1).

### 3.2 Pharmacogenomics of the Serbian population

Pharmacogenetic annotation was performed using PharmCAT (Sangkuhl et al., 2020) and Stargazer (Lee et al., 2019) tools. Differences in haplotype calling were noted between PharmCAT and Stargazer (Table 2). These differences were further inspected using BAM files of subjects. Population pharmacogenomic analysis was performed with results from the annotation tool which gave correct results based on variants found in the BAM file (Table 2). Furthermore, when pharmacogenes harbored important intronic variants that could only be detected with whole exome sequencing, only data from WES were retained for subsequent analysis (Table 1). After removing incorrectly annotated pharmacogenes, Stargazer annotated 170 haplotypes across 47 pharmacogenes, while PharmCAT found 62 star alleles in 13 pharmacogenes (Supplementary Table S2).

**TABLE 2:** Pharmacogenes annotated with PharmCAT or Stargazer based on tool performance after inspecting BAM files. Discordance in haplotype (star allele) calling between PharmCAT and Stargazer is represented in parenthesis (CES | WES).

Optimal annotation – both PharmCAT and Stargazer	Higher accuracy - PharmCAT	Higher accuracy - Stargazer
*ABCG2*		
*CACNA1S*		
*CYP2C9*	*CFTR* (0.3% | 0.0%)	*CYP2B6* (11.1% | 23.0%)
*CYP3A5*	*CYP2C19* (27.5% | 5.3%)	CYP3A4* (NA | 1.8%)
*G6PD*	CYP4F2* (NA | 67.3%)	*DPYD* (14.9% | 3.1%)
*IFNL3*	*RYR1* (100% | 100%)	*SLCO1B1* (8.9% | 0.0%)
*NUDT15*	*UGT1A1* (0.0% | 11.5%)	
*TPMT*		
*VKORC1*		

*Stargazer annotated only *3 and *4 in *CYP4F2* in both CES and WES, while PharmCAT performed poorly in CES, with 232/1536 NA values. Therefore, only WES data was retained and compared between tools. Similarly, in *CYP3A4* both Stargazer and PharmCAT performed poorly for CES data and only WES was retained.

According to Stargazer, the highest number of alternative haplotypes were found in *CYP2D6* (N = 12), whereas the information about *CYP2D6* haplotypes was not available in PharmCAT. PharmCAT reported *RYR1* as the most variable pharmacogene, with 18 alternative haplotypes. Out of 52 analyzed pharmacogenes annotated by either Stargazer or PharmCAT, the highest variability (percent of non-reference haplotypes, or alternative haplotypes) was found for 2 genes–*CYP3A5* (93%) and *CYP2C19* (92%). The least variable pharmacogenes were *CYP26A1* (0.1%), *CFTR* (0.5%), *G6PD* (1.0%), *PTGIS* (1.3%), *NUDT15* (1.3%), *TBXAS1* (3.1%) and *TPMT* (3.3%) (Supplementary Data Sheet 6). Taking into account PGx actionability and frequency in the Serbian population, the most relevant PGx variants are CYP2C9*2, *G6PD* Gond, NAT2*7, SLCO1B1*5, SLCO1B1*14, SLCO1B1*15, UGT1A1*6, UGT1A1*28, UGT1A1*36 and *VKORC1* rs9923231 (Table 4).

After inspecting the results of PGx annotation with PharmCAT and Stargazer, sequencing panels used herein were compared to determine their usefulness in PGx testing (Table 3). For 7 out of 20 pharmacogenes relevant for drug response and therapy optimization based on CPIC guidelines, that were annotated by PharmCAT or Stargazer, the clinical exome sequencing panel gives sufficient information. However, CES cannot capture most of the variants important for drug response in a number of pharmacogenes (13 out of 20). In such cases, whole exome sequencing is needed (Table 3).

**TABLE 3:** Pharmacogenes annotated by PharmCAT or Stargazer that have CPIC published guidelines and can be analysed using clinical exome or whole exome sequencing panels.

Clinical and whole exome sequencing	Whole exome sequencing
	*CACNA1S*
	*CFTR*
	*CYP2B6*
*ABCG2*	*CYP2D6*
*CYP2C8*	*CYP2C19*
*CYP2C9*	*CYP3A4*
*G6PD*	*CYP3A5*
*RYR1*	*CYP4F2*
*SLCO1B1*	*DPYD*
*TPMT*	*IFNL3*
	*NUDT15*
	*UGT1A1*
	*VKORC1*

### 3.3 Comparison between the Serbian and other populations

Overall, 209 star alleles were detected in the Serbian population and their frequencies were further compared to worldwide and European populations. Allele frequencies in the general and European population were extracted from the gnomAD database and from CPIC, while frequencies of PGx haplotypes in the Croatian population were extracted from a previously published study (Matišić et al., 2023). Bonferroni correction for multiple comparison was applied by dividing 0.05 with 52 (number of analyzed genes) and differences between populations were considered significant when p value was <0.0009. Differences in haplotype frequencies were found for 35 out of 52 pharmacogenes.

Compared to the worldwide population, frequencies of 60 star alleles in 31 pharmacogenes were significantly different in the Serbian population, with *SLCO1B1* and *NAT1* harboring the highest number of haplotypes with significantly differing frequencies (higher frequency in the Serbian population: SLCO1B1*5 p < 2.2*10^−16^, SLCO1B1*14 p = 5.2*10^−6^, SLCO1B1*19 p = 1.6*10^−5^, NAT1*15 p = 1.1*10^−6^, NAT1*19 p = 1.8*10^−5^, NAT1*22 p = 1.3*10^−5^; higher frequency in worldwide population: SLCO1B1*15 p = 3.4*10^−7^, SLCO1B1*37 p < 2.2*10^−16^, NAT1*17 p = 7.6*10^−4^).

In comparison to the European population, 48 star alleles in 29 pharmacogenes had statistically significant differences (Supplementary Data Sheets 2–5). Similar to the worldwide population comparison, *NAT1* had the highest number of star alleles with differing frequencies (higher frequency in the Serbian population: NAT1*15 p = 4.2*10^−6^, NAT1*19 p = 8.4*10^−6^, NAT1*22 p = 5.9*10^−5^; higher frequency in European population: NAT1*17 p = 6.31*10^−5^).

Haplotype frequencies were also compared to the Croatian population for 14 pharmacogenes. Due to differences in genotyping panels between the Serbian and the Croatian population, comparisons included only variants covered by both studies. We found differences in star allele frequencies in 4 pharmacogenes between the Serbian and the Croatian group (*CYP2D6, CYP4F2, SLCO1B1* and *UGT1A1*). The most distinctive difference was observed for *SLCO1B1* star alleles showing lower frequencies in the Serbian population (SLCO1B1*5 p < 2.2*10^−16^, SLCO1B1*20 p = 0.006, SLCO1B1*37 p < 2.2*10^−16^).

**FIGURE 1:**
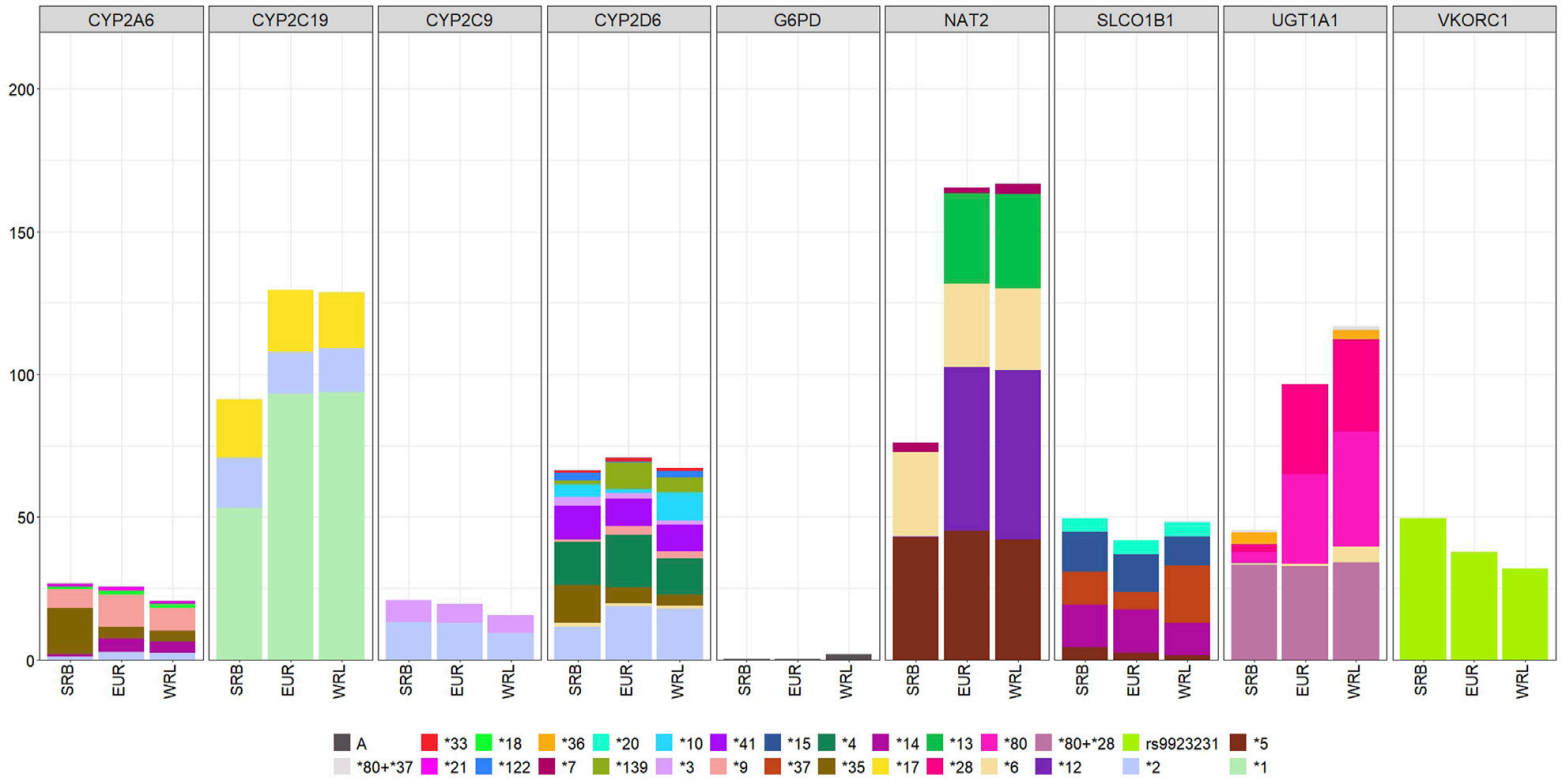
A and B level PGx variants that were detected in the SRB—Serbian population, EUR–European population and WRL–worldwide population.

Star alleles which are considered Level 1 and Level 2 by PharmGKB or level A and B on CPIC and had differing frequencies in Serbian versus other populations are listed in Table 4. Star alleles in level 1/2 and A/B pharmacogenes that had frequencies >1% in the Serbian population and >1% in European and worldwide populations are shown in Figure 1. A complete list of variants with differing frequencies and p values can be found in Supplementary Data Sheets 2, 3, 4, 5.

**TABLE 4:** Star alleles with significantly different frequencies in Serbian compared to worldwide or European populations. Pharmacogenes with the clinical annotation level of evidence 1 and 2 (PharmGKB) or level A and B (CPIC) were included.

Star allele	Frequency in the Serbian population (%)	Frequency in the worldwide population (%)	p value	Frequency in the european population (%)	p value
*CYP2A6* *35	16.4	3.9	<2.2*10^−16^	4.2	<2.2*10^−16^
*CYP2C9* *2	13.2	9.2	1.3*10^−8^	12.8	0.7
*CYP2C19* *1	53.0	93.7	<2.2*10^−16^	93.1	<2.2*10^−16^
*CYP2D6* *35	13.3	3.9	4.01*10^−12^	5.5	5.7*10^−7^
*G6PD* A	0.1	1.7	1.5*10^−7^	0.04	0.5
*G6PD* Gond	0.4	0.04	5.1*10^−6^	0.01	1*10^−11^
*NAT2* *7	3.0	3.4	0.5	1.8	4.5*10^−5^
*NAT2* *12	0.3	59.0	<2.2*10^−16^	57.0	<2.2*10^−16^
*NAT2* *13	0.1	33.0	<2.2*10^−16^	32.0	<2.2*10^−16^
*SLCO1B1* *5	4.2	1.5	<2.2*10^−16^	2.3	3.9*10^−7^
*SLCO1B1* *14	15.0	11.3	5.2*10^−6^	15.3	0.8
*SLCO1B1* *15	14.1	10.2	3.4*10^−7^	13.0	0.2
*SLCO1B1* *37	11.4	20.3	<2.2*10^−16^	6.2	<2.2*10^−16^
*UGT1A1* *6	0.4	5.5	<2.2*10^−16^	0.8	0.3
*UGT1A1* *28	2.7	32.4	<2.2*10^−16^	31.6	<2.2*10^−16^
*UGT1A1* *36	4.1	3.4	0.7	0.0	1.1*10^−8^
*VKORC1* rs9923231	49.6	31.9	1.9*10^−8^	37.8	4*10^−4^

Frequency differences were tested with Chi squared 2 × 2 test or Fisher test. Bonferroni correction for multiple comparisons was applied and differences between used tools as well as between populations were considered significant when p value was <0.0009.

**FIGURE 2:**
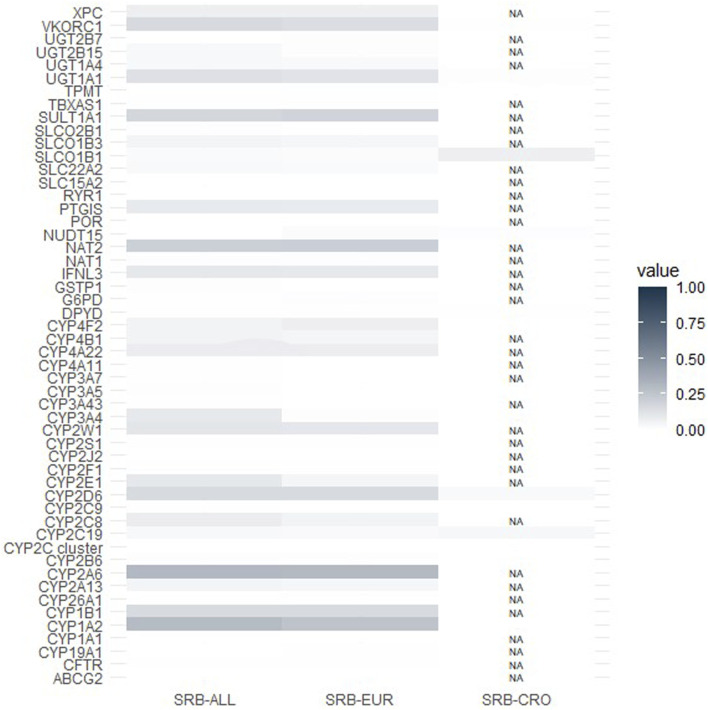
A heatmap representing Wright’s fixation index (Fst). Higher Fst values indicate higher degree of population stratification. NA–no data was available for that pharmacogene in the Croatian population. SRB-ALL–Fst between Serbian and worldwide populations. SRB-EUR–Fst between Serbian and European populations. SRB-CRO–Fst between Serbian and Croatian populations. Star allele frequencies in worldwide and European populations were gathered from CPIC or gnomAD databases, while for Croatia, frequencies were extracted from previously published data (Matišić et al., 2023).

Population similarity between Serbian, worldwide and European populations was further expressed through calculating the Wright’s fixation index (Fst). Values of Fst can range between 0 and 1, with higher values indicating a higher differentiation among populations. Overall, population differentiation was not particularly high for any of the analyzed pharmacogenes (Figure 2), with the highest Fst occurring between the Serbian and the worldwide population for *CYP2A6* (Fst = 0.31). As expected, the Serbian population was more similar to the European than to the worldwide population (mean Fst between worldwide and Serbian populations = 0.052, mean Fst between European and Serbian populations = 0.047). On average, the lowest Fst was found between the Serbian and the Croatian population (p = 0.007).

### 3.4 Pharmacogenetic phenotypes in the Serbian population

Phenotype categories associated with drug categories according to CPIC (level A and B) or PharmGKB (level 1 and 2) were available for 16 out of 52 analyzed pharmacogenes (Table 5). All detected diplotypes alongside predicted phenotypes are listed in Supplementary Data Sheets 7, 8. In accordance with European frequencies, *CYP3A5* poor metabolizers represented 85.8% of the Serbian population, while the rest of tested subjects were intermediate metabolizers (Table 5). Even though carriers of *3/*3 diplotype are characterized as poor metabolizers, they require the standard recommended starting dose, while *1/*1 and *1/*3 (normal and intermediate metabolizer) would require a higher recommended starting dose. The highest prevalence of decreased enzymatic function was found in *NAT2*, with 94.6% of the Serbian population harboring damaging star alleles. The highest percentage of ultrarapid metabolizers was predicted for the *CYP1A2* pharmacogene (84.1%).

**TABLE 5:** Predicted phenotype frequencies of the Serbian population.

Gene	Phenotype	Frequency	Associated drug categories according to CPIC (A/B level) and PharmGKB (1/2 level)
*ABCG2*	Normal function	78.4	Statins
	Decreased function	20.9	
	Poor function	0.7	
*CFTR*	Ivacaftor non-responsive in CF patients	98.8	CFTR modulators
	Ivacaftor responsive in CF patients	1.2	
*CYP1A2*	Normal metabolizers	15.9	NA
	Ultrarapid metabolizers	84.1	
*CYP2A6*	Normal metabolizers	92.0	NA
	Intermediate metabolizers	6.2	
*CYP2A13*	Normal metabolizers	92.1	NA
	Intermediate metabolizers	3.5	
*CYP2B6*	Normal metabolizers	85.0	Antiretrovirals SSRI antidepressants opioids
	Intermediate metabolizers	5.3	
	Ultrarapid metabolizers	4.4	
*CYP2C9*	Normal metabolizers	60.8	NSAIDs statins Anticoagulants Anticonvulsants
	Intermediate metabolizers	36.2	
	Poor metabolizers	3.0	
*CYP2C19*	Normal metabolizers	41.6	Antacids SSRI and SNRI antidepressants antiplatelet medications tricyclic antidepressants antifungals
	Intermediate metabolizers	26.5	
	Rapid metabolizers	23.0	
	Ultrarapid metabolizers	3.5	
	Poor metabolizers	5.3	
*CYP2D6*	Normal metabolizers	54.0	norepinephrine reuptake inhibitors SSRI and SNRI antidepressants antineoplastics opioids antiemetics tricyclic antidepressants antihypertensives
	Intermediate metabolizers	36.3	
	Poor metabolizers	1.8	
*CYP2F1*	Normal metabolizers	41.8	NA
	Intermediate metabolizers	23.8	
	Poor metabolizers	2.5	
*CYP3A5*	Intermediate metabolizers	14.2	immunosuppresants
	Poor metabolizers	85.8	
*CYP3A43*	Normal metabolizers	72.6	NA
	Intermediate metabolizers	8.0	
*CYP4A11*	Normal metabolizers	98.8	NA
	Intermediate metabolizers	1.0	
*CYP19A1*	Normal metabolizers	99.3	NA
	Intermediate metabolizers	0.5	
*DPYD*	Normal metabolizers	95.6	antineoplastics and cytotoxics
	Intermediate metabolizers	4.4	
*G6PD*	Normal function	98.5	antibiotics antimalarials antidotes antigout agents
	Deficient	0.1	
	Variable	0.6	
*IFNL3*	Normal function	49.6	immunomodulators
	Decreased function	40.7	
	No function	9.7	
*NAT2*	Normal function	5.4	antituberculosis agents
	Decreased function	94.6	
*NUDT15*	Normal metabolizers	97.3	immunosuppressants antimetabolites
	Intermediate metabolizers	2.7	
*POR*	Normal function	51.9	NA
	Decreased function	48.0	
	Unknown function	0.1	
*PTGIS*	Normal function	98.2	NA
	Increased function	1.8	
*SLCO1B1*	Normal function	61.5	statins
	Increased function	3.6	
	Decreased function	27.1	
	Poor function	6.5	
*SLCO1B3*	Normal function	3.6	NA
	Decreased function	24.5	
	Poor function	71.9	
*SULT1A1*	Normal function	48.0	NA
	Decreased function	52.0	
*TBXAS1*	Normal function	94.7	NA
	Decreased function	2.7	
*TPMT*	Normal metabolizers	93.6	immunosupressants antimetabolites
	Intermediate metabolizers	4.9	
	Poor metabolizers	0.2	
	Possible intermediate metabolizer	0.1	
*UGT1A1*	Normal metabolizers	34.5	antiretrovirals
	Intermediate metabolizers	46.9	
	Ultrarapid metabolizers	8.8	
*UGT1A4*	Normal function	76.1	NA
	Decreased function	15.9	
*VKORC1*	Normal function	26.5	anticoagulants

NA, pharmacogenes for which level of evidence linking diplotype to phenotype are level 3 and 4 (PharmGKB) or level C and D (CPIC).

**FIGURE 3:**
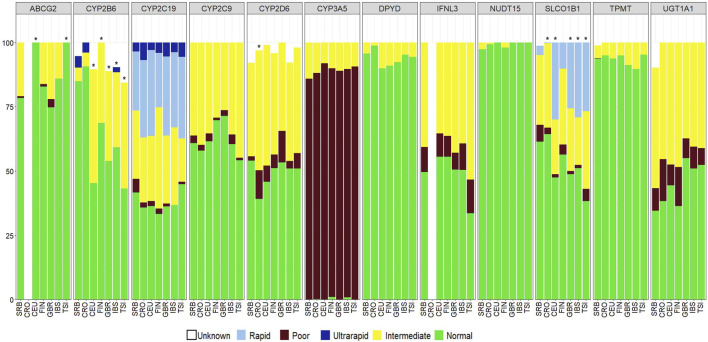
Pharmacogenetic phenotypes in SRB–Serbian, CRO–Croatian, CEU–Central European, FIN–Finnish, GBR–Great Britain, IBS–Iberian (from Spain) and TSI–Tuscan (from Italy) populations. Significance was calculated using Chi squared and Fisher’s Exact tests. After Bonferroni correction for multiple testing, p < 0.004 (SRB-EUR) and p < 0.005 (SRB-CRO) were considered significant.

Comparison of predicted phenotype frequencies between the Serbian and European subpopulations from the 1000 Genomes Project (Central European–CEU, Finnish–FIN, Great Britain–GBR, Tuscans from Italy–TSI and Iberian Spanish–IBS) (Sherman, Claw, and Lee, 2024) was performed for 12 pharmacogenes (*ABCG2, CYP2B6, CYP2C19, CYP2C9, CYP2D6, CYP3A5, DPYD, IFNL3, NUDT15, SLCO1B1, TPMT* and *UGT1A1*) and for 10 pharmacogenes in the Croatian population (*CYP2B6, CYP2C19, CYP2C9, CYP2D6, CYP3A5, DPYD, NUDT15, SLCO1B1, TPMT* and *UGT1A1*) (Supplementary Data Sheet 9). Bonferroni correction for multiple comparison was applied by dividing 0.05 with 12 or 10 for comparisons with European subpopulations or the Croatian population (number of analyzed pharmacogenes) and differences between populations were considered significant when p value was <0.004 or <0.005 respectively. Significant differences between the Serbian and European subpopulations were noted in phenotype distribution for *ABCG2, CYP2B6* and *SLCO1B1* pharmacogenes (Figure 3). The Serbian population had a higher prevalence of decreased function status for the *ABCG2* pharmacogene compared to Central European and Tuscans from Italy (CEU p = 9.1*10^−7^, TSI p = 3.2*10^−7^). European subpopulations had higher frequencies of intermediate metabolizers in *CYP2B6* compared to the Serbian population (CEU p = 9.9*10^−11^, FIN p = 1.6*10^−6^, GBR p = 1.9*10^−7^, IBS p = 6.5*10^−6^, TSI p = 8.1*10^−10^). Increased enzymatic activity of SLCO1B1 was higher in all European subpopulations except in Finnish compared to the Serbian population (CEU p = 4.6*10^−13^, GBR p = 4.0*10^−10^, IBS p = 6.2*10^−14^, TSI p = 1.4*10^−11^). Significant differences between the Serbian and the Croatian population were noted for poor metabolizers in *CYP2D6*, which were more frequent in the Croatian population (p = 0.003) and for poor and rapid metabolizers in *SLCO1B1*, with the Serbian population having higher frequencies of both poor and rapid metabolizers (poor metabolizers p = 0.0008, rapid metabolizers p = 8.9*10^−5^). Other pharmacogenes that were analyzed in this part of the study had phenotype frequencies in accordance with European subpopulations and the Croatian population.

## 4 Discussion

Fast development of NGS technologies revolutionized genomic research and clinical diagnostics. Efforts for implementing NGS into therapy optimization are noted globally, with many working groups gathering to upgrade pharmacogenomics recommendations for drug usage (CPIC, DPWG). This study presents the first comprehensive analysis of star allele frequencies in more than 50 pharmacogenes in the Serbian population from a large group of subjects previously analyzed using NGS, enhancing the knowledge related to potentially important and population specific PGx variants that could be considered as markers for preemptive and predictive PGx testing in the Serbian population.

One of the objectives of this study was to determine whether clinical exome sequencing (CES) and whole exome sequencing (WES) data could be effectively utilized for pharmacogenomics profiling. For 29 analyzed pharmacogenes, variants were successfully annotated regardless of which panel was used for sequencing, indicating applicability of both CES and WES technologies in pharmacogenomic testing. Out of those 29 pharmacogenes, 7 are included in the CPIC guidelines for drug dosing (i.e., *ABCG2, SLCO1B1, CYP2C8, CYP2C9, G6PD, RYR1* and *TPMT*). This could be important since CES is a more cost-effective alternative to WES for comprehensive genomic testing and could be considered a reliable approach for extensive pharmacogenetic analysis. However, when it comes to overall PGx profiling, WES proved to be more informative, additionally covering all relevant pharmacogenes and PGx variants (CYP2C19*17, CYP3A5*3, *NUDT15, UGT1A1* TATA box, *VKORC1*). Nevertheless, for detecting complex structural rearrangements, whole genome sequencing (WGS) is the method of choice. This is particularly important for genotyping of *CYP2D6* – in our study, we identified only single nucleotide variants and small indels, even though it would be expected to find at least a few copy number variants in our sample of several hundred individuals. Therefore, we conclude that WGS should be required for precise profiling of *CYP2D6*. Nonetheless, we showed that employing CES and WES to clinical pharmacogenomics is feasible for many pharmacogenes, especially for those included in published guidelines for drug dose individualization according to patient’s genotype.

Besides testing usability of the applied NGS platforms, we also tested performance of the two most commonly employed tools for annotating pharmacogenomic variants, PharmCAT and Stagazer. Calling of star alleles with both PharmCAT and Stargazer was performed for 18 pharmacogenes. We noticed discordant results in almost half of the analyzed pharmacogenes between these two tools. Discrepancies were mostly related to the possibly outdated or incomplete data of relevant pharmacogenetic variants in Stargazer (particularly variants in *RYR1* and variant 711 + 3A>G in *CFTR*) or PharmCAT (variants in *DPYD*). Furthermore, inconsistencies between tools were found in the annotation of pharmacogenes that harbored complex haplotypes, where haplotype phasing is dubious without case-parent triads. Since we used unphased genomic data of unrelated individuals, retained diplotypes were the ones that were most probable based on frequencies in the European population. Overall, we determined that using more than one annotation tool for PGx studies is indeed adequate, with additional manual inspection and rigorous quality control to resolve discrepancies between the outputs of different tools.

We further analyzed distribution of pharmacogenetic variants in the Serbian population and compared it to other populations. The most important pharmacogenes detected in the Serbian population, that harbored actionable variants with notable frequency and are included in the CPIC guidelines were *CYP2C9, NAT2, SLCO1B1, UGT1A1* and *VKORC1.* These pharmacogenes could be considered for inclusion into genetic reports, since they affect response to many drugs (anticoagulants, antituberculosis agents, statins, immunosuppresive and anticancer drugs). Significant differences in star allele frequencies were identified in 31, 29, and 4 pharmacogenes when compared to worldwide, European and Croatian populations, respectively. Most of these differences were observed in pharmacogenes with low levels of PGx evidence. However, 12 pharmacogenes showing differences between the Serbian population and worldwide or European populations are classified as Level A or B according to the CPIC guidelines. These include *CFTR, CYP2C9, CYP2C19, CYP2D6, CYP4F2, G6PD, NAT2, RYR1, SLCO1B1, TPMT, UGT1A1* and *VKORC1*. Compared to the worldwide population, the Serbian population exhibits significantly higher frequencies of specific A or B level star alleles (CYP2D6*35, *RYR1* c.4178A>G, *RYR1* c.10042C>T) as well as lower frequencies of others (CYP2C19*1, *G6PD* A, NAT2*12, NAT2*13, *RYR1* c.10747G>C, SLCO1B1*37). Similar trends were observed when compared with the European population, except that the SLCO1B1*37 variant was additionally found as more frequent, while variants CYP2C19*1, NAT2*12, NAT2*13, *RYR1* c.10747G>C were found as less frequent in the Serbian population. These alleles determine the normal or uncertain function of the encoded proteins. Star alleles that influence PGx phenotype and have a higher frequency in the Serbian population compared to other Europeans are NAT2*7, SLCO1B1*5, UGT1A1*36 and *VKORC1* -1639A, while UGT1A1*28 had a lower frequency in the Serbian population.

One of the variants that had significantly higher frequency in the Serbian population compared to both worldwide and European population was 711 + 3A>G in *CFTR* gene. Since genomic data used in our study was from individuals subjected to exome sequencing for diagnostic purposes, we cannot exclude a possibility that in our group some individuals had a diagnosis of cystic fibrosis–hence the higher frequency of a pathogenic variant in our sample.

Moreover, our study showed a significantly higher frequency of *VKORC1* -1693A in the Serbian population compared to worldwide and European populations, which is in alignment with previously published data (Kovac et al., 2010). Furthermore, previous results of higher incidence of CYP2C9*3 in the Serbian population (Mizzi et al., 2016), differs from our findings. Said star allele had a frequency in alignment with the European population. As for thiopurine drugs, association studies of drug response and PGx variants were previously conducted on pediatric patients with acute lymphoblastic leukemia and adult patients with inflammatory bowel disease in Serbia. In these studies, frequencies of PGx variants in *TPMT* (Dokmanovic et al., 2006; Jojic et al., 2003)*, TYMS, SLC19A11, DHFR, ITPA, ABCC4* and *ABCB1* (Milosevic et al., 2018) were reported. Adding to variants TPMT*2, TPMT*3A and TPMT*3C that were previously reported in the Serbian population, this study detected three more star alleles in *TPMT* – *9, *20 and *43. Frequency of SLCO1B1*5 was previously reported in the Serbian population as well (Kotur et al., 2020). Notably, previous studies report SLCO1B1*5, associated with response to methotrexate, as more frequent than in our results. We detected SLCO1B1*5 as a singular variant and as a part of a haplotype SLCO1B1*15. If we were to combine the frequencies of the variant and the haplotype it constitutes, SLCO1B1*5 would have a similar frequency to the one previously reported. Previous studies had limited samples and used PCR-based genotyping followed with Sanger sequencing or genotyping arrays (Kovac et al., 2010; Mizzi et al., 2016; Kotur et al., 2020; Dokmanovic et al., 2006; Milosevic et al., 2018). Even though most of these studies were conducted on patients with different diagnoses, variants in pharmacogenes are not considered disease causing, hence these results could reflect the genuine PGx profile of the Serbian population. Comparison of our results with previous findings outlines the need for PGx testing prior to the introduction of treatment to different diseases.

In pharmacogenes *CACNA1S*, *CYP17A1* and *CYP2R1* only reference alleles were found in the Serbian population. This finding was as expected, since alternative alleles present in position files for star allele calling with Stargazer and PharmCAT are considered rare in the rest of the world as well.

Furthermore, an analysis of population differentiation through Wright’s fixation index calculation showed that the Serbian population exhibited the greatest dissimilarity from the global population, particularly for *CYP2A6* (Fst = 0.31). As anticipated, the Serbian and the Croatian population showed the highest level of similarity.

Due to availability of data and geographical proximity in the Western Balkan region, the Croatian population was included in this study for fine-tuning comparison. Population data was also published for the Slovenian population (Hočevar, Maver, and Peterlin, 2019). Owing to different statistical approaches used in our and Slovenian group, further comparisons were not anaysed between the two populations. Frequencies of most of the analyzed star alleles were concordant between two populations. This finding is further corroborated with Wright’s fixation index calculation that showed high genetic similarity between Serbian and Croatian population. The highest difference was found for SLCO1B1*5, SLCO1B1*37 and CYP4F2*3 which were more frequent in the Croatian population. We cannot exclude that this discrepancy might be due to different methodological approaches in the two compared studies–we used clinical and whole exome sequencing, a methodology that covers wider regions of the genome. The group from Croatia used a targeted panel that covers only the most important star alleles, thus other alternative alleles might have been missed in their sample. Moreover, higher frequency in the Croatian population was similar as previously reported for the Serbian population for UGT1A1*28 star allele (Vukovic et al., 2018). The discrepancy that was presented herein is probably due to a different methodological approach–in our study we detected UGT1A1*28 in two forms, as an individual variant and as part of a haplotype (UGT1A1*80+*28). By merging frequencies of UGT1A1*28 and UGT1A1*80+*28, the frequency of UGT1A1*28 would be similar to previously reported data in the Serbian and in the Croatian population.

A significant part of our study represents analysis of PGx phenotypes related to several drug categories in the Serbian population. Many of these phenotypes determine protein function, but there is still a lack of evidence as to how changes in protein structure affect drug metabolism. Therefore, further comparisons between Serbian and other European subpopulations were conducted only for pharmacogenes included in guidelines published by relevant consortia, such as CPIC. We found that a significant portion of the Serbian population (94.5%) carries variants in *NAT2* (*5, *6 and *7) that induce decreased enzymatic activity and could contribute to drug induced liver injury in patients treated with isoniazid (Ng et al., 2014). Significant discrepancy was also found in star alleles influencing response to statin therapy, namely, in phenotypes determined by *ABCG2* and *SLCO1B1*. We found that *ABCG2* intermediate statin metabolizers were more frequent in the Serbian population than in Central European and Tuscans. In contrast, *SLCO1B1* rapid metabolizers were less frequent in the Serbian population. Hepatic uptake of statins is regulated via a transporter encoded by *SLCO1B1*, while absorption and disposition are modified by a *ABCG2*-encoded efflux transporter (Rhonda et al., 2022). Even though *SLCO1B1* rapid metabolizers were less frequent in the Serbian population compared to European populations, this does not imply that testing for *SLCO1B1* irregular responders would be superfluous. Intermediate and poor responders were as expected compared with other Europeans, however, they represented over 20% of our sample. Furthermore, in the Serbian population, *CYP2B6* intermediate metabolizers, characterized by reduced enzymatic activity, were more frequent compared to all other analysed subpopulations. Several drugs from the antidepressant category, such as anxiolytics, are thought to be influenced by *CYP2B6* phenotype (Radosavljevic et al., 2023). Such findings in our population indicate that the Serbian population may benefit from the introduction of genotyping of patients for variants in *NAT2*, *ABCG2*, *SLCO1B1* and *CYP2B6* prior to administration of antituberculosis agents, statins and antidepressants due to differences that were presented herein.

The main limitation of our study was the number of samples sequenced with clinical exome compared to the whole exome panel. For most of the analyzed pharmacogenes, WES proved to be more efficient, due to the inclusion of many important PGx variants in splicing regions and exons that are not covered by the CES panel. Even though WES provided us with a large number of variants, for pharmacogenomics studies, whole genome sequencing would be even more informative. For example, we detected many star alleles in *CYP2D6*, but we were not able to detect any copy number variants. Moreover, tools for annotation of star alleles that we used in our study do not annotate a highly polymorphic *HLA* gene, which plays an important role in response to antiepileptic and antiretroviral therapeutics. Another limitation of our study was that data was de-identified and any tracking of diagnosis, congenital anomaly or therapy response was unfeasible. Furthermore, several databases were used for allele frequency comparisons and Wright’s fixation index calculation. Ideally, all data would be compared to one database with matched sample sizes, however, no such database exists to the best of our knowledge. Frequencies of PGx variants can be found in PharmGKB, but originate from several databases: CPIC, All of US, UK Biobank, gnomAD, 1000 Genomes Project. Frequencies throughout databases should not differ significantly, however, we cannot exclude that the use of several sources affected results presented in our study. An important limitation in population genomic studies is the presence of biases in worldwide datasets, which can significantly influence comparisons using Wright’s fixation index (Fst). The 1000 Genomes project and gnomAD data are skewed towards European ancestry with many global populations still underrepresented. Also, sample size disparities introduce higher variance in allele frequency estimates; if one population has large sample size while other have small, Fst values could be misleading. Furthermore, many datasets lump genetically distinct subpopulations into broad categories and if these subpopulations are not equally represented, intra-population heterogeneity can reduce observed Fst values, masking true differentiation. Therefore, these limitations should be carefully considered when interpreting population differences, particularly when making comparisons against broad, worldwide datasets.

In conclusion, this study represents an in-depth analysis of the pharmacogenomics landscape of the Serbian population and our sample constitutes the largest group of individuals ever analyzed in a PGx study in Serbia. We used data from several databases (CPIC, 1,000 Genome Project, gnomAD, published studies) to compare our findings to other populations. Moreover, we summarized the PGx phenotype distinctions of our population considering different therapeutics that are used in Serbia as well. Our findings reveal interethnic heterogeneity in key pharmacogenes, such as *NAT2*, *SLCO1B1*, *UGT1A1* and *VKORC1* and offer valuable insights into the applicability of various sequencing technologies and variant annotation tools in PGx analysis. We showed that results often depend on used methodology and annotation tools. Therefore, careful selection of pharmacogenes, star alleles, sequencing panels and annotation tools is advised for PGx implementation. Furthermore, the conducted study is the representation of PGx phenotypes specific to the Serbian population, supporting the integration of relevant pharmacogenomic information into genetic reports tailored for this population.

## Data Availability

The original contributions presented in the study are included in the article/Supplementary Material, further inquiries can be directed to the corresponding author.
