# Supplementary material for: Comprehensive pharmacogenomics profiling of the Serbian population
Source: Front Pharmacol. 2025 Mar 17;16:1553536. doi: 10.3389/fphar.2025.1553536 (PMC11955590; doi:10.3389/fphar.2025.1553536)
Supplement: Supplementary file 1 [file Table1.docx]

Table S1: Pharmacogenes withdrawn from subsequent analysis based on Hardy-Weinberg Equilibrium

| Clinical exome sequencing panel (CES) | Whole exome sequencing panel (WES) | CES+WES | Pharmacogenes in which only the referent allele was present |
| --- | --- | --- | --- |
| *p* value < 0.001 | *p* value < 0.0009 | *p* value < 0.002 |  |
| *ABCB1*  *CYP2A6*  *CYP2D6*  *CYP3A43*  *UGT2B15* | *ABCB1*  *GSTM1*  *SLCO1B1*  *SULT1A1* | *ABCB1*  *CYP3A43*  *UGT2B15* | *CACNA1S*  *CYP17A1*  *CYP2R1* |

*p* value of Hardy-Weinberg equilibrium after Bonfferoni correction for multiple testing was used as a measure of variant annotation quality

Table S2: Star alleles (pharmacovariants or haplotypes) found in Serbian population

| Pharmacogene | Stargazer | PharmCAT |
| --- | --- | --- |
| *ABCG2* | g.88131171G>T (p.Q141K) | g.88131171G>T (p.Q141K) |
| *CFTR* |  | g.117611595T>G (p.F1052V)  g.117509089C>T (p.R74W)  g.117540284C>T (p.R352W)  g. 117534368A>G (711+3A>G) |
| *CYP2C* cluster | g.94645745G>A |  |
| *CYP1A1* | *2C (g.74720644T>C)  *4 (g.74720646G>T)  *5(g.74720638G>T) |  |
| *CYP1A2* | *9 (g.74749986C>T)  *1F (g.74749576C>A)  *1K (g.74749576C>A, g.74749000T>G, g.74749010C>T) |  |
| *CYP1B1* | *2 (g.38075034C>A, g.38075247:G>C)  *3 (g.38071060G>C)  *4 (g.38070996T>C)  *6 (g.38075034C>A, g.38075247:G>C, g.38071060G>C)  *7 (g.38075034C>A, g.38075247:G>C, g.38071060G>C, g.38071026G>C) |  |
| *CYP2A6* | *2 (g.40848628A>T)  *9 (g.40850474A>C)  *14 (g.40850341C>T)  *17 (g.40845362C>T)  *18 (g.40844759T>A)  *21 (g.40843854T>C)  *35 (g.40843969T>A) |  |
| *CYP2A13* | *2 (g.41088545G>A, g.41091846C>T)  *3 (g.41090100G>GCCA, g.41090177C>G)  *7 (g.41089049C>T)  *8 (g.41090177C>G) |  |
| *CYP2B6* | *2 (g.40991369C>T, p.R22C)  *4 (g.41009358A>G, p.K262R)  *5 (g.41016810C>T, p.R487C)  *6 (g.41009358A>G, g.41006936G>T, p.K262R+p.Q172H)  *9 (g.41006936G>T, p.Q172H)  *11 (g.40991441A>G, p.M46V)  *13 (g.41004377A>G, g.41009358A>G, g.41006936G>T, p.K139E+p.K262R+p.Q172H)  *14 (g.41004381G>A, p.R140Q)  *22 (g.40991224T>C, c.-82T>C) |  |
| *CYP2C8* | *2 (g.95058349T>A)  *3 (g.95067273C>T, g.95038992T>C)  *4 (g.95058362G>C) |  |
| *CYP2C9* | *2 (g.94942290C>T, p.R144C)  *3 (g.94981296A>C, p.I359L)  *11 (g.94981224C>T, p.R335W)  *12 (g.94989020C>T, p.P489S)  *29 (g.94972119C>A, p.P279T)  *35 (g.94942290C>T, g.94942234G>T, p.R144C+p.R125L)  *67 (g.94988852C>T, p.R433W) | *2 (g.94942290C>T, p.R144C)  *3 (g.94981296A>C, p.I359L)  *11 (g.94981224C>T, p.R335W)  *12 (g.94989020C>T, p.P489S)  *29 (g.94972119C>A, p.P279T)  *35 (g.94942290C>T, g.94942234G>T, p.R144C+p.R125L)  *67 (g.94988852C>T, p.R433W |
| *CYP2C19* |  | *1 (g.94842866A>G, p.I331V)  *2 (g.94775367A>G, g.94775507G>A, g.94781859G>A, g.94842866A>G, c.12662A>G+p.R150H+c.19154G>A+p.I331V)  *4 (g.94842866A>G, g.94762706A>G, g.94761900C>T,  c.-806C>T+p.I331V+p.M1V)  *8 (g.94842866A>G, g.94775416T>C, p.I331V+p.W120R)  *17 (g.94842866A>G, g.94761900C>T,  c.-806C>T+p.I331V) |
| *CYP2D6* | *2 (g.42127941G>A, g.42126611C>G, p.R296C+p.S486T)  *3 (g.42128241CT>C, p.R259fs)  *4 (g.42128945C>T, g.42129130C>G, c.1847G>A+c.1662G>C)  *6 (g.42129083CA>C, p.W152fs)  *9 (g.42128173CCTT>C, p.K281del)  *10 (g.42130692G>A, g.42129130C>G, p.P34S+c.1662G>C)  *33 (g.42128308C>A, p.A237S)  *35 (g.42129130C>G, g.42127941G>A, g.42126611C>G, g.42130761C>T, c.1662G>C+p.R296C+ p.S486T+p.V11M)  *41 (g.42127803C>T, c.2989G>A)  *59 (g.42127852C>T, c.2940G>A)  *122 (g.42127512C>T, p.V370I)  *139 (g.42127526C>T, p.R365H) |  |
| *CYP2E1* | *4 (g.133532171G>A)  *5 (g.133526101G>C, g.133526341C>T)  *7 (g.133527063T>A) |  |
| *CYP2F1* | *2 (g.41116202G>GC)  *3 (g.41121963G>A, g.41122109G>C, g.41128075C>T)  *4 (g.41116300T>C, g.41121963G>A)  *5 (g.41125512T>C)  *6 (g.41116576G>C) |  |
| *CYP2J2* | *7 (g.59926822C>A) |  |
| *CYP2S1* | *2 (g.41203611C>T)  *3 (g.41206370C>T) |  |
| *CYP2W1* | *2 (g.985219G>A)  *6 (g.988812C>T) |  |
| *CYP3A4* | *1B (g.99784473T>C)  *7 (g.99778079C>T)  *8 (g.99770165C>T)  *22 (g.99768693G>A) |  |
| *CYP3A5* | *3 (g.99672916T>C, c.6981A>G) | *3 (g.99672916T>C, c.6981A>G) |
| *CYP3A7* | *1D (g.99735184C>T)  *2 (g.99709062G>C) |  |
| *CYP3A43* | *1B (g.99861633C>T)  *2A (g.99836454TA>T)  *2B (g.99836454TA>T. g.99859982C>G)  *3 (g.99859982C>G) |  |
| *CYP4A11* | g.46932824A>G (p.F434S)  g.46934207T>C (p.S353G) |  |
| *CYP4A22* | *2 (g.47142179A>T, g.47143817T>C, g.47137516C>T)  *3 (g.47144551TG>T)  *5 (g.47142179A>T, g.47143817T>C)  *9 (g.47142179A>T, g.47143817T>C, g.47141609C>T, g.47145926T>C)  *12 (g.47142179A>T, g.47143817T>C, g.47141609C>T, g.47145926T>C, g.47142113G>A, g.47143311G>T)  *15 (g.47142179A>T, g.47143817T>C, g.47141609C>T, g.47145926T>C, g.47142113G>A, g.47143311G>T, g.47144393A>C, g.47148762C>T) |  |
| *CYP4B1* | *2 (g.46815074GAT>G)  *3 (g.46813503C>T)  *4 (g.46815158A>G)  *5 (g.46815187G>A) |  |
| *CYP4F2* |  | *3 (g.15879621C>T, p.V433M)  *4 (g.15897578A>C, g.15879621C>T, p.W12G+p.V433M)  *5 (g.15878779G>T, p.L519M)  *6 (g.15890405C>A, p.G185V)  *14 (g.15886018G>C, p.L341V)  *17 (g.15878920T>C, p.T472A) |
| *CYP19A1* | *3 (g.51222375G>A)  *4 (g.51215771G>A) |  |
| *CYP26A1* | *4 (g.93076616T>C) |  |
| *DPYD* | g.97450058C>T (1905+1G>A, *2A)  g.97515865C>T (1601G>A, *4)  g.97515839T>C (1627A>G, *5)  g.97305364C>T (2194G>A, *6)  g.97883329A>G (85T>C, *9A)  g.97579893G>C, g.97573863C>T (1129-5923C>G, 1236G>A, HapB3)  g.97699535T>C (496A>G)  g.97450068A>G (1896T>C)  g.97679170T>C (775A>G)  g.97699408C>A (623G>T) |  |
| *G6PD* | A (g.154535277T>C, p.N126D)  Mediterranean,Dallas,Panama,Sassari,Cagliari (g.154534419G>A, p.S188F)  Andalus (g.154532389C>T, p.R454H)  Seattle,Lodi,Modena,Ferrara-II,Athens-like (g.154533596C>G, p.D282Y)  Gond (g.154535176C>G, p.M159I) | A (g.154535277T>C, p.N126D)  Mediterranean,Dallas,Panama,Sassari,Cagliari (g.154534419G>A, p.S188F)  Andalus (g.154532389C>T, p.R454H)  Seattle,Lodi,Modena,Ferrara-II,Athens-like (g.154533596C>G, p.D282Y)  Gond (g.154535176C>G, p.M159I) |
| *GSTP1* | *B (g.67585218A>G, p.I105V)  *C (g. 67585218A>G, g.67586108C>T, pI105V+pA114V)  *D (g.67586108C>T, pA114V) |  |
| *IFNL3* | g.39241143A>G (c.-1595G>A) | g.39241143A>G (c.-1595G>A) |
| *NAT1* | *10 (g.18223135T>A, g.8223142C>A)  *14 (g.18222607G>A)  *15 (g.18222606C>T)  *17 (g.18222237C>T)  *19 (g.18222144C>T)  *22 (g.18222799A>T) |  |
| *NAT2* | *5 (g.18400344T>C)  *6 (g.18400593G>A)  *7 (g.18400860G>A)  *10 (g.18400502G>A)  *12 (g.18400806A>G)  *13 (g.18400285C>T)  *14 (g.18400194G>A) |  |
| *NUDT15* | *3 (g.48045719C>T, p.R139C) | *3 (g.48045719C>T, p.R139C) |
| *POR* | *5 (g.75983548G>C)  *16 (g.75985795G>A)  *28 (g.75985688C>T)  *45 (g.75980462G>A) |  |
| *PTGIS (CYP8A1)* | *1D (g.49568117C>CGCGGGGCTG)  *1E (g.49568117C>CGCGGGGCTGGCGGGGCTG) |  |
| *RYR1* |  | g.38492540G>T (c.6178G>T)  g.38486015C>T (c.5360C>T)  g.38534726C>G (c.11266C>G)  g.38483293A>G (c.4711A>G)  g.38519292G>A (c.10097G>A)  g.38543551A>G (c.11798A>G)  g.38475335A>G (c.4178A>G)  g.38566986G>C (c.13513G>C)  g.38519237C>T (c.10042C>T)  g.38496265C>T (c.6599C>T)  g.38516167A>G (c.9635A>G)  g.38527707G>C (c.10747G>C)  g.38455247A>G (c.1453A>G)  g.38494379T>A (c.6302T>A)  g.38499241A>G (c.7025A>G)  g.38485691G>A (c.5036G>A)  g.38502652A>G (c.7760A>G)  g.38496901G>A (c.6838G>A) |
| *SLC15A2* | *2 (g.121924957C>T, g.121928439C>T, g.121929321G>A) |  |
| *SLC22A2* | *2 (g.160258368C>A)  *3 (g.160224800T>C)  *6 (g.160249250C>A) |  |
| *SLCO1B1* | *5 (g.21178615T>C, p.V174A)  *14 (g.21176879C>A, g.21176804A>G, p.P155T+p.N130D)  *15 (g.21178615T>C, g.21176804A>G,p.V174A+ p.N130D)  *19 (g.21239042A>C, p.L643F)  *20 (g.21178615T>C, g.21239042A>C, p.V174A+ p.L643F)  *37 (g.21176804A>G, p.N130D)  *45 (g.21222355C>T, p.R580X)  *46 (g.21178615T>C, g.21176804A>G, g.21222355C>T, p.V174A+ p.N130D+p.R580X) |  |
| *SLCO1B3* | g.20862826G>A |  |
| *SLCO2B1* | g.75162709GCACAGAAAA>G  g.75196537C>T |  |
| *SULT1A1* | *2 (g.28606193C>T) |  |
| *TBXAS1* | *2 (g.139872324G>A)  *3 (g.139953397C>A)  *7 (g.140015841G>A)  *8 (g.140015845C>A) |  |
| *TPMT* | *2 (g.18143724C>G, p.A80P)  *3A (g.18138997C>T, g.18130687T>C, p.A154T+p.Y240C)  *3C (g.18138997C>T, p.A154T)  *9 (g.18143606T>G, p.K119T)  *20 (g.18130694T>C, p.K238E)  *43 (g.18143700C>T, g.18143728C>A, p.G88S+p.W78C) | *2 (g.18143724C>G, p.A80P)  *3A (g.18138997C>T, g.18130687T>C, p.A154T+p.Y240C)  *3C (g.18138997C>T, p.A154T)  *9 (g.18143606T>G, p.K119T)  *20 (g.18130694T>C, p.K238E)  *43 (g.18143700C>T, g.18143728C>A, p.G88S+p.W78C) |
| *UGT1A1* |  | *6 (g.233760498G>A, p.G71R)  *28 (TA8)  *36 (TA6)  *80 (g.233759924C>T, c.-364C>T)  *80+*37 (c.-364C>T+TA9)  *80+*28 (C.-364C>T+TA8) |
| *UGT1A4* | *2 (g.233718890C>A)  *3A (g.233718602C>T, g.233718658G>A, g.233718962T>G) |  |
| *UGT2B7* | *2 (g.69098620C>T)  *3 (g.69096731G>T) |  |
| *UGT2B15* | *2 (g.68670366C>A)  *4 (g.68647129T>G)  *5 (g.68670366C>A, g.68647129T>G)  *6 (g.68655133G>A) |  |
| *VKORC1* | g.31096368C>T (c.-1639G>A) | g.31096368C>T (c.-1639G>A) |
| *XPC* | g.14145949G>T  g.14158387G>A |  |

Genomic position of pharmacovariants is written as in GRCh38 genome assembly
